# Medial Prefrontal and Anterior Insular Connectivity in Early Schizophrenia and Major Depressive Disorder: A Resting Functional MRI Evaluation of Large-Scale Brain Network Models

**DOI:** 10.3389/fnhum.2016.00132

**Published:** 2016-03-30

**Authors:** Jacob Penner, Kristen A. Ford, Reggie Taylor, Betsy Schaefer, Jean Théberge, Richard W. J. Neufeld, Elizabeth A. Osuch, Ravi S. Menon, Nagalingam Rajakumar, John M. Allman, Peter C. Williamson

**Affiliations:** ^1^Department of Psychiatry, University of Western Ontario, LondonON, Canada; ^2^Imaging Division, Lawson Health Research Institute, LondonON, Canada; ^3^First Episode Mood and Anxiety Program, London Health Sciences Centre, LondonON, Canada; ^4^Department of Medical Biophysics, University of Western Ontario, LondonON, Canada; ^5^Department of Psychology, University of Western Ontario, LondonON, Canada; ^6^Neuroscience Program, University of Western Ontario, LondonON, Canada; ^7^Centre for Functional and Metabolic Mapping, Robarts Research Institute, University of Western Ontario, LondonON, Canada; ^8^Department of Anatomy and Cell Biology, University of Western Ontario, LondonON, Canada; ^9^Division of Biology, California Institute of Technology, PasadenaCA, USA; ^10^Tanna Schulich Chair in Neuroscience and Mental Health, Department of Psychiatry, University Hospital, London Health Sciences Centre, LondonON, Canada

**Keywords:** anterior insula, functional network connectivity, major depressive disorder, medial prefrontal cortex, resting fMRI, schizophrenia

## Abstract

Anomalies in the medial prefrontal cortex, anterior insulae, and large-scale brain networks associated with them have been proposed to underlie the pathophysiology of schizophrenia and major depressive disorder (MDD). In this study, we examined the connectivity of the medial prefrontal cortices and anterior insulae in 24 healthy controls, 24 patients with schizophrenia, and 24 patients with MDD early in illness with seed-based resting state functional magnetic resonance imaging analysis using Statistical Probability Mapping. As hypothesized, reduced connectivity was found between the medial prefrontal cortex and the dorsal anterior cingulate cortex and other nodes associated with directed effort in patients with schizophrenia compared to controls while patients with MDD had reduced connectivity between the medial prefrontal cortex and ventral prefrontal emotional encoding regions compared to controls. Reduced connectivity was found between the anterior insulae and the medial prefrontal cortex in schizophrenia compared to controls, but contrary to some models emotion processing regions failed to demonstrate increased connectivity with the medial prefrontal cortex in MDD compared to controls. Although, not statistically significant after correction for multiple comparisons, patients with schizophrenia tended to demonstrate decreased connectivity between basal ganglia-thalamocortical regions and the medial prefrontal cortex compared to patients with MDD, which might be expected as these regions effect action. Results were interpreted to support anomalies in nodes associated with directed effort in schizophrenia and nodes associated with emotional encoding network in MDD compared to healthy controls.

## Introduction

Frith (1992) proposed that schizophrenia was associated with anomalies in connections between the medial prefrontal cortex, the dorsolateral prefrontal cortex, and anterior cingulate cortex. His proposal was that these aberrant connections lead to a disorder of agency. That is, the positive and negative symptoms of schizophrenia reflect impairments in the perception and initiation of action. When we are *directing effort*, we realize that we are controlling our internal thoughts. Patients with schizophrenia often feel that they are not in control and that someone is putting thoughts in their head or their thoughts are heard as someone else’s voice- a hallucination. Subsequent brain imaging studies have confirmed that activity in the medial prefrontal cortex is associated with many aspects of directed effort such as the ability to represent the thoughts, feelings, and actions of self and others across time, known as mentalizing (Gilbert et al., 2006). Thus aberrant connectivity between the medial prefrontal cortex and other brain regions might be expected in patients with schizophrenia.

Mood disorders have also been associated with anomalies in the connections between the medial prefrontal cortex and other parts of the brain. However, in contrast to schizophrenia, ventral medial prefrontal regions have been implicated. In a comprehensive review, Phillips et al. (2003a,b) concluded that mood disorders were associated with changes in brain activity in the ventral anterior cingulate cortex and subgenual prefrontal cortex and their connections with other emotional processing regions including the amygdala, insula, and ventral striatum.

The anterior insulae have been implicated in both schizophrenia and mood disorders as well. The anterior insulae participate in a number of functions including decision making, the re-representation of interoception and sudden insight (Craig, 2009). Seeley et al. (2007) demonstrated that the anterior insulae are closely connected to the dorsal anterior cingulate cortex forming a *salience network* that directs attention to relevant environmental cues. Anomalies in the salience network have been suggested to underlie many of the imaging findings in patients with schizophrenia (Palaniyappan and Liddle, 2012) and other major psychiatric disorders (Menon, 2011).

In recent years, a number of large-scale networks involving both the medial prefrontal cortex and anterior insulae have been implicated in both schizophrenia and major depressive disorder (MDD) (Williamson, 2007; Williamson and Allman, 2012; Kaiser et al., 2015; Mulders et al., 2015). Menon (2011) proposed that most major psychiatric disorders could be accounted for by aberrant intrinsic organization and interconnectivity of the salience network, the default mode network (DMN) and the central executive network. In schizophrenia, structural and functional anomalies were proposed to affect all three networks whereas in MDD, excessive coupling between the salience network and the DMN was proposed.

Buckner et al. (2008) proposed two brain systems, the DMN and a medial temporal lobe network. The DMN was suggested to be associated with internally focused tasks, autobiographical memory, envisioning the future, and conceiving the perspective of others. The medial temporal lobe system was suggested to be associated with information about prior experience, which is used for mental simulations in the medial regions. Information from both networks was suggested to converge through the posterior cingulate cortex and schizophrenia was proposed to be associated with over-activity of the DMN.

Northoff and Qin (2011) proposed a somewhat different model for schizophrenia, where auditory verbal hallucinations were seen as arising from elevated resting state activity in the auditory cortex possibly related to abnormal modulation of the auditory cortex by midline structures associated with the DMN. Core systems for self-representation in MDD were suggested to be ‘highjacked’ by lower sub-cortical primary-process emotional systems (Northoff et al., 2011). When this happens, negative thoughts such as feeling worthless or hopeless might be enhanced in depressed patients. More recent reviews of the literature have generally supported increased connectivity between the anterior DMN and the salience network and/or emotion processing regions of the brain in MDD (Kaiser et al., 2015; Mulders et al., 2015).

Williamson and Allman (2011, 2012) argued that structures such as the frontal pole, temporal pole, and anterior insula are highly developed in humans and likely to be associated with the representation of the thoughts, feelings, and actions of self and others across time. These representational nodes interact with *dorsal* directed effort regions including the dorsal anterior and posterior cingulate cortices, the auditory cortex, and the hippocampus and *ventral* emotional encoding regions including the orbital prefrontal cortex, the ventral anterior cingulate cortex, and the amygdala. Failure of the directed effort nodes was proposed to be associated with schizophrenia while failure of the emotional encoding nodes was proposed to underlie mood disorders.

Although, there have been a number of studies of resting networks in each patient group, we are not aware of any studies that have evaluated the connectivity of the medial prefrontal cortex and anterior insulae in both patient groups early in illness. The objective of this study was to evaluate predictions that the proposed large-scale network models would make about connectivity in these regions in healthy controls and patients with schizophrenia or MDD early in illness.

With regard to medial prefrontal connectivity, Williamson and Allman (2011, 2012) would predict that the medial prefrontal cortex would have reduced connectivity with nodes associated with directed effort including the dorsal anterior and posterior cingulate cortices, and temporal regions in patients with schizophrenia while patients with MDD would have reduced connectivity between the medial prefrontal cortex and ventral emotional encoding regions. Buckner et al. (2008) and Northoff and Qin (2011) would predict decreased connectivity between the medial prefrontal cortex and posterior cingulate cortex and temporal regions in patients with schizophrenia. Northoff et al. (2011) would predict increased connectivity between emotion processing regions and the medial prefrontal cortex in MDD. With regard to anterior insulae connectivity, increased connectivity between the anterior insulae and the medial prefrontal cortex would be predicted by Menon (2011) in MDD. Widespread anterior insulae connectivity deficits particularly with regions associated with executive control would be predicted by Menon (2011), Williamson and Allman (2011, 2012), and Palaniyappan and Liddle (2012) in patients with schizophrenia.

## Materials and Methods

### Participants

Approval for the protocol was obtained from the research ethics board at the University of Western Ontario. After a complete description of the study to the participants, written informed consent was obtained. Twenty-four patients with schizophrenia and 24 patients with MDD, within approximately 2 years of diagnosis, along with 24 healthy controls of comparable age, handedness, and parental education level, assessed as in our previous study (Aoyama et al., 2011), were recruited from the community and through the Prevention and Early Intervention in Psychosis and First Episode Mood and Anxiety Programs in London, London, ON, Canada. All patients and controls were assessed by an experienced rater (B.S.) and psychiatrist (P.W.) with the Structured Clinical Interview for DSM-IV (SCID; First et al., 1997) to establish a consensus diagnosis of schizophrenia or MDD and exclude psychiatric diagnoses in healthy volunteers. Symptoms were rated immediately prior to the functional magnetic resonance imaging (fMRI) scanning with the Scale for the Assessment of Positive Symptoms (SAPS; Andreasen, 1984), the Scale for the Assessment of Negative Symptoms (SANS; Andreasen, 1984) in patients with schizophrenia and the Montgomery-Asberg Depression Scale (Montgomery and Asberg, 1979) in patients with MDD. Demographic data are shown in **Table 1**. Patients or controls with a history of drug or alcohol abuse in the previous year, mental retardation, hypertension, diabetes, hepatic/renal insufficiency, or neurological conditions were excluded. Controls with a known family history of psychiatric disorder in a first- or second-degree relative were excluded. All patients with schizophrenia had no history of a major depressive episode.

**Table 1:** Participant demographic information.

	Schizophrenic patients (*n* = 24) mean (SD)	Major depressive disorder patients (*n* = 24) mean (SD)	Healthy controls (*n* = 24) mean (SD)
Age, years	23.2 (4.2)	21.2 (4.3)	23.8 (4.3)
Gender, male/female	21/3	8/16	12/12
Handedness, right/left	23/1	21/3	21/3
Parental education level^a^	3(1)	3(1)	3(1)
Smoking status^b^, yes/no	13/11	2/22	0/24
Illness duration^c^, range	13.7 (10.9) 2–36	13.5 (10.4) 3–45	–
SANS, mean	22.5 (14.5)	–	–
SAPS, mean	10.3 (11.9)	–	–
Montgomery-Asberg, mean	–	22.7 (8.5)	–
On neuroleptics, atypical/typical/none	21/2/1	1/0/23	–
On antidepressants, yes/no	–	16/8	–
CPZ eq, mg/day	258.0 (222.5)	–	–


*SD, Standard deviation; SANS, Scale for the Assessment of Negative Symptoms; SAPS, Scale for the Assessment of Positive Symptoms; CPZ eq, chlorpromazine equivalent dose.*

*^a^1, grade 10 or below; 2, grade 11–13; 3, college/university 1–3 years; 4, college/university 4 years or more.*

*^b^Smoking status obtained by self-report.*

*^c^Illness duration: in months from first psychotic symptom in schizophrenic patients and from diagnostic threshold level met in MDD patients.*

### Resting Functional Magnetic Resonance Imaging Acquisition

Magnetic resonance imaging (MRI) data were acquired using a 3.0 Tesla MRI scanner (Siemens Tim Trio, Erlangen, Germany) at the Centre for Functional and Metabolic Mapping (CFMM; Robarts Research Institute, University of Western Ontario), using a 32-channel head coil. Whole-brain T1-weighted anatomical images with 1 mm isotropic resolution were acquired as reference for spatial normalization of the data. Functional MRI scans consisted of a 2D multi-slice, gradient-echo, echo-planar (T2^∗^-weighted) scan (TR = 3 s, TE = 20 ms, flip angle = 90°, field of view = 256 mm × 256 mm × 120 mm, 10 min scan time for 200 volumes plus two discarded steady state volumes per run, parallel to the AC-PC plane) covering the whole-brain with an isotropic resolution of 2 mm.

During the resting scan participants were instructed to keep their eyes closed and let their minds wander without falling asleep; all reported they were able to comply with the instructions after the scan.

### fMRI Data Preprocessing

All fMRI data underwent standard preprocessing steps. The first three volumes of the fMRI scan were discarded to ensure T1 signal equilibrium had been reached. Each image volume was then realigned to the first volume based on six movement parameters (translation in x, y, z and rotation in yaw, pitch, roll; INRIAlign^1^) to correct for motion. The fMRI images were then normalized into the standard Montreal Neurological Institute space using the corresponding T1-weighted anatomical images, and smoothed with a 10 mm full width at half-maximum 3D Gaussian kernel (SPM8^2^). ARtifact detection Tools (ART^3^) was used to detect movement (>2 mm) and mean global image intensity outliers in the fMRI data and to generate a corresponding movement and artifact multiple regressor file for each subject. The multiple regressor file contained the six realignment parameter regressors and a seventh regressor for flagged outliers; subjects with greater than two flagged outliers were excluded from the analysis, which included two SZ patients. Finally, all fMRI data were band-pass filtered (0.012–0.1 Hz) to extract the blood-oxygenation level dependant (BOLD) signals.

### First-Level fMRI Data Analysis

A first-level, within-subject, seed-based connectivity analysis (SPM8^2^), as in Bluhm et al. (2009), was performed and involved entering 10 mm radius spherical seed regions positioned in the left insula (-40, 18, -4; MNI coordinates), right insula (40, 18, -4; MNI coordinates), and the medial prefrontal cortex (-1, 49, -2; MNI coordinates) as regressors of interest. The multiple regressor files that were calculated for each subject during preprocessing (INRIAlign) were entered as regressors of no interest to mitigate residual movement and artifacts. The resulting positive correlation t-maps for each subject, which represent the correlation strength of each voxel to the seed region, were carried forward to the group-wise analysis.

### Second-Level fMRI Data Analysis

To address our objectives, we examined within-group connectivity patterns [ANOVA, *n* = 72, df = 67, false-discovery rate (FDR) peak-level corrected *p* < 10^-13^] and between-group connectivity differences (*post hoc* pair-wise *t*-tests, *n* = 72, df = 67, FDR, *p* < 0.05) to the medial prefrontal cortex and left and right insulae (SPM8^2^), using the correlation t-maps generated in the first-level analysis. Gender and self-reported smoking status were included as covariates of no interest in all group-wise comparisons to help mitigate the clear gender and smoking status differences between groups.

## Results

### Clinical Measures

Chlorpromazine equivalent levels, SANS, SAPS, and Montgomery-Asberg scores did not correlate with connectivity between the fronto-insular seeds and medial prefrontal seed and other brain regions.

### Resting Functional Connectivity

**FIGURE 1:**
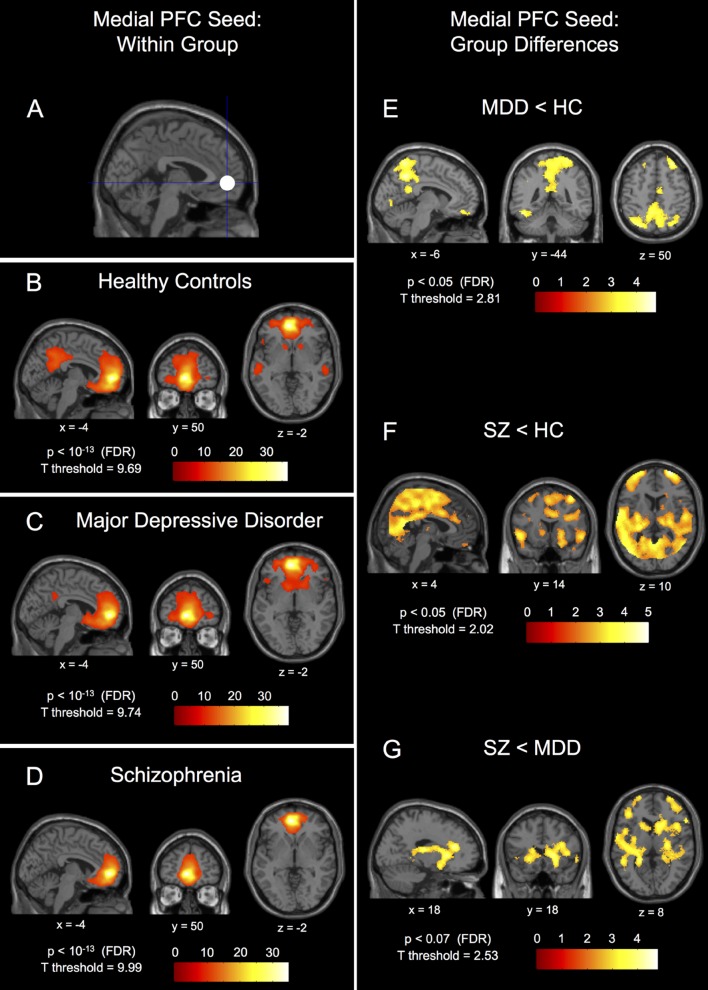
**Within-group connectivity to the medial prefrontal cortex seed and between-group differences.** The 10 mm radius medial prefrontal cortex (PFC) seed (-1, 49, -2) shown in **(A)** and it’s within-group connectivity shown below for healthy controls (HC) in **(B)**, major depressive disorder patients (MDD) in **(C)**, and schizophrenic patients (SZ) in **(D)**. Group differences in connectivity are shown for MDD < HC in **(E)**, SZ < HC in **(F)**, and SZ < MDD in **(G)**. Seed coordinates and image labels are in MNI space. All *p*-value thresholds were set for display purposes only.

**FIGURE 2:**
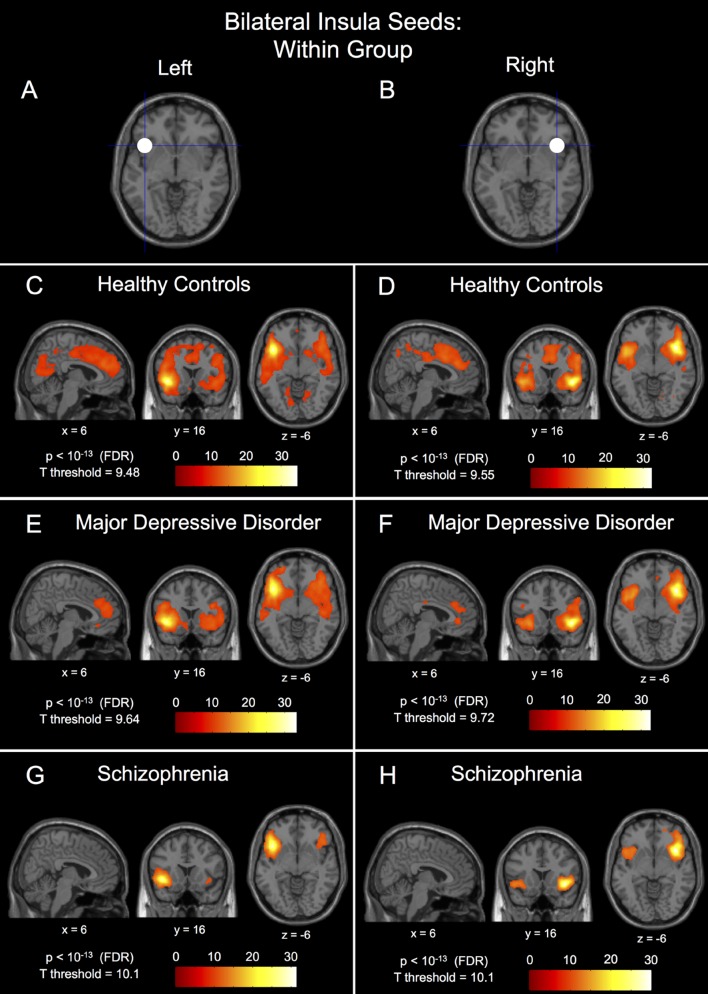
**Within-group connectivity to the bilateral insula seeds in healthy controls, major depressive disorder patients, and schizophrenic patients.** The 10 mm radius left insula seed centered at (-40, 18, -4) shown in **(A)** and it’s within-group connectivity shown below for healthy controls in **(C)**, major depressive disorder patients in **(E)**, and schizophrenic patients in **(G)**. The 10 mm radius right insula seed centered at (40, 18, -4) shown in **(B)** and it’s within-group connectivity shown below for healthy controls in **(D)**, major depressive disorder patients in **(F)**, and schizophrenic patients in **(H)**. Seed coordinates and image labels are in MNI space. All *p*-value thresholds were set for display purposes only.

We identified within-group connectivity patterns for each group for the medial prefrontal seed (**Figures 1A–D**) and for the left and right insula seeds (**Figures 2A–H**), results overlaid on normalized, T1-weighted, anatomical images. We identified a statistically significant main effect of group for both the left and right insulae and the medial prefrontal seeds (*p* < 0.05, FDR-corrected).

#### Within-Group ANOVA

The within-group patterns of connectivity to the medial prefrontal seed are shown in **Figures 1B–D** showing drastically different connectivity to the posterior cingulate cortex; strong connectivity in healthy controls, reduced connectivity in patients with MDD, and no significant connectivity to the posterior cingulate cortex in patients with schizophrenia. The three groups also showed markedly different within-group patterns of connectivity for both the left and right insula seeds; healthy controls showed large clusters of connectivity to the dorsal anterior cingulate cortex with some posterior clusters whereas patients with MDD showed only a small dorsal anterior cingulate cortex cluster and patients with schizophrenia showed no connectivity to the dorsal anterior cingulate cortex whatsoever (**Figures 2C–H**). Significant within-group clusters of connectivity for each seed for all three groups are listed in **Table 2**.

**Table 2:** Clusters of within-group seed connectivity.

Region	BA	*k^a^*	Peak MNI coordinate (x, y, z)	Peak *T*-score^b^	*p*-value (FDR-corrected)
**Medial PFC seed**					
**Healthy controls**					
L,R medial frontal gyrus	10	13454	-4, 50, -2	36.58	0.000
L,R cingulate gyrus	31	2717	-2, -50, 40	14.27	0.000
L middle temporal gyrus	21	789	-58, -18, -12	13.50	0.000
R middle temporal gyrus	21	300	50, -8, -22	11.93	0.000
L inferior frontal gyrus	47	228	-46, 20, -10	11.77	0.000
L superior parietal, precuneus	7	486	-24, -76, 46	11.75	0.000
R superior temporal gyrus	39	555	48, -54, 24	11.45	0.000
R inferior frontal gyrus	47	166	38, 30, -20	11.14	0.000
R superior temporal gyrus	22	200	60, -28, -2	10.80	0.000
**Major depressive disorder patients**					
L,R medial frontal gyrus	10	14646	-4, 50, -2	38.19	0.000
L middle temporal gyrus	21	336	-62, -22, -10	12.67	0.000
R inferior frontal gyrus	45	92	56, 26, 6	11.61	0.000
L,R cingulate gyrus	31	85	-4, -44, 34	10.68	0.000
**Schizophrenic patients**					
L,R medial frontal gyrus	10	5580	-4, 50, -2	34.70	0.000
***Right insula seed***					
**Healthy controls**					
R,L inferior frontal, insula	13/47	33071	40, 18, -6	32.15	0.000
L middle occipital gyrus	19	72	-34, -76, 16	11.04	0.000
L superior parietal, precuneus	7	242	-14, -64, 50	10.76	0.000
R superior parietal lobule	7	70	14, -50, 74	10.44	0.000
R occipital, lingual gyrus	19	109	22, -66, -10	10.29	0.000
**Major depressive disorder patients**					
R inferior frontal, insula	13/47	11622	40, 18, -6	32.35	0.000
L inferior frontal, insula	13/47	3038	-36, 10, -4	16.34	0.000
L middle frontal gyrus	10	1335	-36, 48, 14	13.25	0.000
L middle cingulate gyrus	23	75	-2, -16, 30	11.48	0.000
R anterior cingulate gyrus	24	147	4, 38, 0	11.16	0.000
R superior frontal gyrus	6	51	36, -8, 46	10.40	0.000
**Schizophrenic patients**					
R Interior frontal, insula	13/47	2899	40, 18, -6	29.82	0.000
L inferior frontal, insula	13/47	817	-40, 6, -2	13.34	0.000
R parietal gyrus	7/40	134	40, -40, 40	11.33	0.000
R superior frontal gyrus	10	95	34, 42, 22	11.12	0.000
L superior frontal gyrus	10	55	-28, 46, 22	11.04	0.000
R superior frontal gyrus	6	50	40, 4, 46	10.63	0.000
**Left insula seed**					
**Healthy controls**					
L,R inferior frontal, insula	13/47	44405	-42, 18, -4	34.47	0.000
R occipital, lingual gyrus	19	345	18, -64, -10	11.44	0.000
**Major depressive disorder patients**					
L,R inferior frontal, insula	13/47	23153	-42, 20, -4	33.10	0.000
**Schizophrenic patients**					
L inferior frontal, insula	13/47	3040	-42, 20, -4	31.27	0.000
R inferior frontal, insula	13/47	565	46, 34, -10	13.15	0.000


*BA, Brodmann area; k, cluster size; L, left; MNI, standard Montreal Neurological Institute space; PFC, prefrontal cortex; R, right.*

*^a^Cluster threshold = 50, *p* < 10^*–13*^ (false-discovery rate corrected).*

*^b^*T*-scores with degrees of freedom (1,67).*

#### *Post hoc* Between-Group Analysis

Results of *post hoc* between-group comparisons are listed in **Table 3**; with Brodmann area (BA), cluster size, peak coordinates, *T*-score, FDR-corrected *p*-value, and uncorrected *p*-value for significant clusters of connectivity. Note that there were no significant differences in connectivity to any of the three seeds for comparisons not reported in **Table 3** (i.e., MDD > Controls, etc.).

**Table 3:** Clusters of between-group seed connectivity differences.

Region	BA	*k*^a^	Peak MNI coordinate (x, y, z)	Peak *T*-score^b^	FDR corrected *p*-value^c^	Uncorrected *p*-value
**Medial PFC seed**						
**Major Depressive Disorder < Controls**						
L fusiform gyrus	37	1657	-36, -58, -16	4.75	0.037	0.000
L,R superior parietal, precuneus	7	10846	-8, -56, 44	4.65	0.037	0.000
L orbitofrontal gyrus (vmPFC)	11	107	-6, 44, -16	3.55	0.037	0.000
R fusiform gyrus	37	466	30, -58, -14	3.53	0.037	0.000
R inferior frontal gyrus, orbital	47	85	36, 28, -24	3.23	0.038	0.001
L superior frontal gyrus	8	189	-18, 28, 58	3.21	0.038	0.001
L post-central gyrus	1	227	-34, -36, 68	3.17	0.039	0.001
**Schizophrenia < Controls**						
R superior frontal/R,L parietal	10/7	40568	28, 68, 8	4.97	0.010	0.000
L superior frontal gyrus	9	1172	-18, 60, 32	3.99	0.010	0.000
L dorsal ACC	32	162	-12, 34, 20	3.66	0.010	0.000
L orbitofrontal gyrus (vmPFC)	11	99	-10, 48, -22	3.63	0.010	0.000
R dorsal ACC	32	151	14, 34, 20	3.55	0.010	0.000
R superior temporal gyrus	22	307	56, 8, -6	3.40	0.010	0.001
L middle temporal gyrus	21	53	-36, -4, -18	3.32	0.011	0.001
R inferior frontal gyrus, orbital	47	286	20, 18, -20	3.25	0.011	0.001
R superior frontal gyrus	10/9	75	12, 62, 28	3.21	0.011	0.001
**Schizophrenia < Major Depressive Disorder**						
R dorsal ACC/caudate/thalamus/putamen	32	5532	16, 32, 18	4.78	0.062	0.000
L posterior insula/putamen	13	5235	-34, -26, 2	4.35	0.062	0.000
R middle-lateral frontal gyrus	10	887	42, 48, 2	3.65	0.062	0.000
L superior frontal gyrus (dlPFC)	10	611	-28, 64, 18	3.61	0.062	0.000
R medial frontal gyrus (vmPFC)	10	55	12, 44, -6	3.33	0.062	0.001
R post-central gyrus	1	95	46, -20, 32	3.26	0.062	0.001
R fusiform gyrus	37	69	50, -50, -8	3.12	0.062	0.001
**R insula seed**						
**Major Depressive Disorder < Controls**						
L fusiform gyrus	37	1601	-34, -54, -16	4.92	0.014	0.000
R fusiform gyrus	37	962	34, -60, -14	4.90	0.014	0.000
R,L superior parietal, precuneus	7	16131	22, -44, 78	4.87	0.014	0.000
L temporal pole	38	940	-44, 16, -26	4.09	0.014	0.000
L superior temporal gyrus	22/39	127	-58, -42, 18	3.83	0.015	0.000
R posterior cingulate gyrus	23/29	150	8, -50, 10	3.34	0.020	0.001
R superior frontal gyrus	9	66	18, 52, 40	3.11	0.025	0.001
**Schizophrenia < Controls**						
R,L superior parietal, precuneus	7	21256	26, -46, 74	4.57	0.018	0.000
L superior temporal gyrus	41	1498	-50, -24, 2	4.48	0.018	0.000
L temporal pole	38	62	-36, 8, -38	3.87	0.018	0.000
R middle frontal gyrus	10	53	28, 68, 8	3.57	0.018	0.000
L posterior insula/putamen	13	86	-28, -18, 6	3.56	0.018	0.000
R dorsal ACC	32	139	16, 32, 24	3.39	0.019	0.001
R middle frontal gyrus	9/8	231	50, 14, 36	3.34	0.019	0.001
L temporal pole	38	89	-44, 16, -22	3.20	0.021	0.001
R superior frontal gyrus	9	57	12, 60, 40	3.18	0.021	0.001
L middle frontal gyrus	6	59	-24, 10, 68	3.10	0.023	0.001
L superior frontal gyrus	9	84	-26, 54, 40	3.06	0.023	0.002
**Schizophrenia < Major Depressive Disorder**						
L temporal lobe	21/22	193	-38, -44, 6	4.37	0.543	0.000
R parietal lobe	40	619	42, -38, 24	4.26	0.543	0.000
R dorsal ACC	32	107	16, 32, 18	3.79	0.543	0.000
L post-central gyrus	1	161	-36, -20, 28	3.61	0.543	0.000
L posterior-lateral thalamus	–	115	-22, -26, 10	3.25	0.543	0.001
R superior frontal gyrus (dlPFC)	10	100	42, 54, 16	3.10	0.543	0.001
**L insula seed**						
**Major Depressive Disorder < Controls**						
L,R superior parietal, precuneus	7	7990	-24, -72, 48	4.54	0.050	0.000
R fusiform gyrus	37/19	806	24, -60, -10	4.52	0.050	0.000
L fusiform gyrus	37	1276	-36, -58, -14	4.35	0.050	0.000
L superior frontal gyrus	8	441	-20, 28, 58	4.16	0.050	0.000
L temporal pole	38	214	-48, 16, -22	4.07	0.050	0.000
R temporal pole	38	233	40, 20, -30	4.01	0.050	0.000
R superior frontal gyrus	8	426	32, 34, 54	3.98	0.050	0.000
R middle cingulate gyrus	24	65	10, -14, 44	3.64	0.050	0.000
L,R middle cingulate gyrus	24/32	209	0, 8, 38	3.57	0.050	0.000
L hippocampus/parahippocampus	35	90	-22, -16, -16	3.47	0.050	0.000
R posterior cingulate gyrus	23/29	100	10, -50, 12	3.41	0.050	0.001
R middle frontal gyrus	8	87	48, 10, 42	3.34	0.050	0.001
L superior temporal gyrus	22	50	-60, -44, 16	3.34	0.050	0.001
L middle frontal gyrus	8	88	-46, 10, 46	3.34	0.050	0.001
**Schizophrenia < Controls**						
R,L superior parietal, precuneus	7	31657	30, -74, 42	5.28	0.005	0.000
R middle temporal gyrus	21/22	335	48, 0, -20	4.16	0.005	0.000
L middle frontal gyrus	8	1059	-24, 28, 46	4.03	0.005	0.000
L superior frontal gyrus (dlPFC)	9	273	-30, 32, 20	3.74	0.006	0.000
L middle temporal gyrus	21	132	-60, -34, -12	3.61	0.007	0.000
R middle frontal gyrus	10	89	26, 68, 10	3.58	0.007	0.000
L superior frontal gyrus (dlPFC)	9	112	-20, 54, 34	3.21	0.011	0.001
R temporal pole	38	50	26, 18, -30	3.04	0.014	0.002
**Schizophrenia < Major Depressive Disorder**						
R superior temporal gyrus	22	1665	42, -40, 22	4.58	0.141	0.000
L insula	13	1369	-40, 4, 18	4.23	0.141	0.000
R dorsal ACC	32	214	14, 32, 20	3.95	0.141	0.000
L superior temporal gyrus	41	423	-50, -20, 4	3.88	0.141	0.000
R anterior putamen	–	264	24, 16, 8	3.87	0.141	0.000
L superior temporal gyrus	41/22	205	-44, -40, 10	3.75	0.141	0.000
R posterior cingulate gyrus	23/31	334	8, -56, 22	3.35	0.141	0.001
R medial frontal gyrus	10	223	22, 52, 10	3.16	0.144	0.001
R medial frontal gyrus (vmPFC)	11	77	10, 32, -10	3.13	0.147	0.001
L medial frontal gyrus	10	134	-28, 36, 20	3.05	0.149	0.002


*ACC, Anterior cingulate cortex; BA, Brodmann area; dlPFC, dorsolateral PFC; FDR, False-Discovery Rate; k, cluster size; L, left; MNI, standard Montreal Neurological Institute space; PFC, prefrontal cortex; R, right; vmPFC, ventral-medial PFC.*

*^a^Cluster threshold = 50.*

*^b^*T*-scores with degrees of freedom (1,67).*

*^c^All clusters with *p* < 0.005 (uncorrected) peak-level were included.*

#### Analysis of the Medial Prefrontal Cortex

*Post hoc* between-group comparisons in the medial prefrontal seed network showed differences between healthy controls and patients with MDD (**Figure 1E**), MDD having significantly reduced connectivity to bilateral fusiform gyrus (BA37), bilateral parietal gyrus/precuneus (BA7), left orbitofrontal gyrus (BA11), right inferior frontal gyrus (BA47), left superior frontal gyrus (BA8), and left post-central gyrus (BA1). Patients with schizophrenia had reduced medial prefrontal cortex seed connectivity compared to healthy controls to bilateral parietal gyrus/precuneus (BA7), right superior frontal gyrus (BA10/9), left superior frontal gyrus (BA9), bilateral dorsal anterior cingulate gyrus (BA32), left orbitofrontal gyrus (BA11), right superior temporal gyrus (BA22), left middle temporal gyrus (BA21), and right inferior frontal gyrus (BA47; **Figure 1F**). There were no differences in connectivity to the medial prefrontal cortex seed between patients with schizophrenia and MDD that met FDR-corrected significance at *p* < 0.05, however, there was a trend toward reduced connectivity (*p* = 0.062, FDR-corrected) in patients with schizophrenia compared to patients with MDD to the right dorsal anterior cingulate gyrus (BA32), right caudate, right thalamus, right putamen, left posterior insula (BA13), left putamen, right middle-lateral and ventral-medial frontal gyri (BA10), left superior frontal gyrus (BA10), right post-central gyrus (BA1), and right fusiform gyrus (BA37; **Figure 1G** – *p* < 0.07, FDR-corrected).

#### Analysis of the Anterior Insulae

**FIGURE 3:**
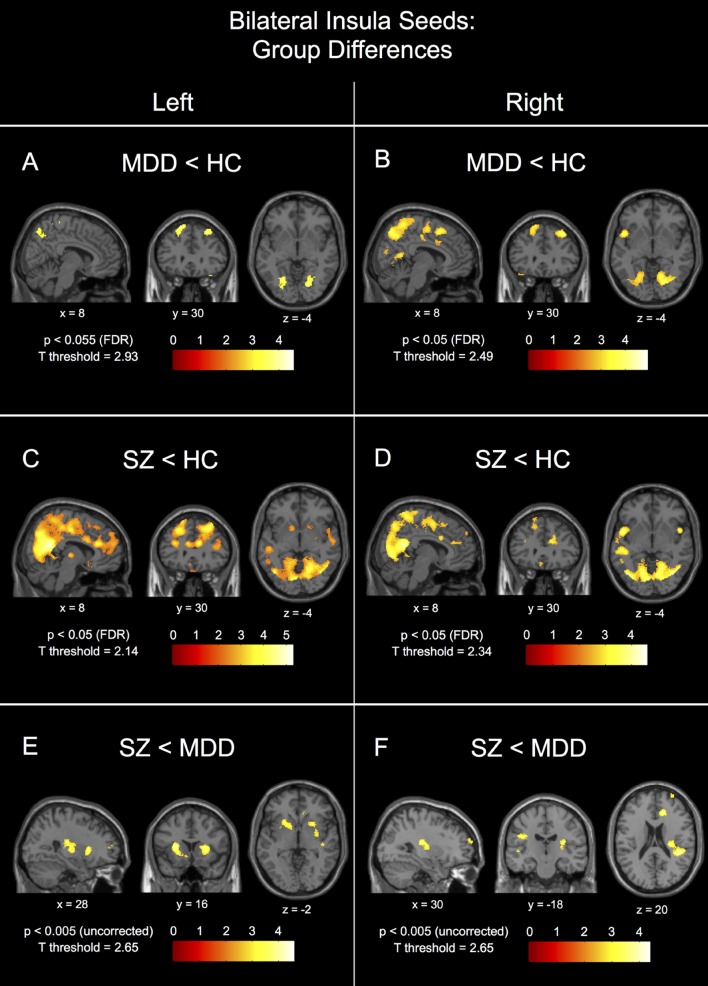
**Between-group connectivity differences for the bilateral insula seeds.** Group differences are shown for the left insula seed for major depressive disorder patients (MDD) < healthy controls (HC) in **(A)**, schizophrenic patients (SZ) < HC in **(C)**, and SZ < MDD in **(E)**. Group differences are shown for the right insula seed for MDD < HC in **(B)**, SZ < HC in **(D)**, and SZ < MDD in **(F)**. Each image is labeled with its MNI coordinate. All *p*-value thresholds were set for display purposes only.

*Post hoc* between-group comparisons in the left insula seed network showed differences between healthy controls and patients with MDD (**Figure 3A**), MDD having significantly reduced connectivity to bilateral parietal gyrus/precuneus (BA7), fusiform gyrus (BA37/19), superior and middle frontal gyri (BA8), temporal pole (38), and middle cingulate gyrus (24/32), and hippocampus/parahippocampal gyrus (BA35), right posterior cingulate gyrus (BA23/29), and left superior temporal gyrus (BA22). Patients with schizophrenia had reduced left insula seed connectivity compared to healthy controls to a number of parietal regions with peaks in bilateral parietal gyrus/precuneus (BA7) and middle temporal gyrus (BA21/22), left middle frontal gyrus (BA8), left superior frontal gyrus (BA9), right middle frontal gyrus (BA10), and right temporal pole (BA38), and with large clusters of voxels extending into the middle and anterior cingulate cortices (**Figure 3C**). There were no FDR-corrected significant differences in connectivity to the left insula seed between patients with schizophrenia and MDD, however, patients with schizophrenia had reduced uncorrected left insula seed connectivity compared to patients with MDD to bilateral superior temporal gyrus (BA22/41), left insula (BA13), right dorsal anterior cingulate gyrus (BA32), right anterior putamen, right posterior cingulate cortex (BA23/31), bilateral medial frontal gyrus (BA10), and right medial frontal gyrus (BA11; **Figure 3E** – *p* < 0.005, uncorrected).

*Post hoc* between-group comparisons in the right insula seed network showed differences between healthy controls and patients with MDD (**Figure 3B**), MDD having significantly reduced connectivity to bilateral fusiform (BA37), bilateral parietal gyrus/precuneus (BA7), left temporal pole (BA38), left superior temporal gyrus (BA22/39), right posterior cingulate gyrus (BA23/29), and right superior frontal gyrus (BA9). Patients with schizophrenia had reduced right insula seed connectivity compared to healthy controls to a number of parietal regions with peaks in bilateral precuneus (BA7), left superior temporal gyrus (BA41), left temporal pole (BA38), right middle frontal gyrus (BA10/9/8), left posterior insula/putamen (BA13), right dorsal anterior cingulate gyrus (BA32), bilateral superior frontal gyrus (BA9), and left middle frontal gyrus (BA6; **Figure 3D**). There were no FDR-corrected significant differences in connectivity to the right insula seed between patients with schizophrenia and MDD, however, patients with schizophrenia had reduced uncorrected right insula seed connectivity compared to patients with MDD to left temporal gyrus (BA21/22), right parietal gyrus (BA40), right dorsal anterior cingulate gyrus (BA32), left post-central gyrus (BA1), left posterior-lateral thalamus, and right superior frontal gyrus (BA10; **Figure 3F**).

Interestingly, patients with schizophrenia showed patterns of connectivity for the left and right insula seeds that were different than MDD to the posterior and dorsal anterior cingulate regions (**Figures 3A–D**).

## Discussion

### Medial Prefrontal Seed Connectivity in Patients with Schizophrenia

#### Within Group Comparisons

Within group connectivity showed significantly reduced functional connectivity between the medial prefrontal cortex and the posterior cingulate cortex and other regions of the DMN in patients with schizophrenia in contrast to healthy controls and depressed patients who demonstrated varying degrees of connectivity.

#### Control Comparisons

In keeping with our hypotheses, decreased connectivity between a seed in the medial prefrontal cortex and many of the nodes associated with directed effort, including the dorsal anterior and posterior cingulate cortices, superior temporal cortex and dorsolateral prefrontal cortex were found in early schizophrenia compared to controls. This is consistent with the model of Williamson and Allman (2012) and a recent study in drug-naïve patients (Cui et al., 2015).

The decreased connectivity of the medial prefrontal seed with the orbital frontal cortex in schizophrenic patients was unexpected. However, the dorsal anterior cingulate cortex and ventral prefrontal cortex are anti-correlated (Mayberg et al., 1999). There is considerable evidence that the dorsal anterior cingulate cortex is overactive early in schizophrenia (Williamson and Allman, 2011, 2012). Thus over-activity in the dorsal anterior cingulate cortex in schizophrenia may lead to decreased activity in the ventral anterior cingulate cortex, which can then lead to decreased connectivity with the medial prefrontal cortex and perhaps account for affective symptoms such as flat affect and poor motivation. We did not find a correlation between ventral cingulate cortex activity and negative symptoms in our patients but this might be related to the fact that they were early in illness and had relatively low ratings of negative symptoms.

### Medial Prefrontal Seed Connectivity in MDD

#### Control Comparisons

Decreased connectivity was found between the medial prefrontal seed and a ventral mood processing region in early MDD compared to controls consistent with the Williamson and Allman (2012) model and consistent with a meta-analysis of resting state data in depression at different ages (Kaiser et al., 2015). There was also reduced connectivity between the medial prefrontal seed and posterior regions of the DMN in patients with MDD compared to healthy controls.

#### Comparisons to Patients with Schizophrenia

Compared to MDD, patients with schizophrenia tended to have decreased connectivity between the medial prefrontal cortex and widespread regions including the dorsal anterior cingulate cortex and regions associated with the basal ganglia-thalamocortical (BGTC) circuit. Although these differences just failed to make the FDR correction, BGTC circuits are closely associated with directed effort. The basal ganglia receive projections from the prefrontal cortex and then project via the thalamus back to the cortex to allow highly specific and co-ordinated control of neural activity. Five BGTC circuits were originally characterized for cognitive, affective, and motor control (Alexander et al., 1990).

Aberrant topography of the intrinsic connectivity of the striatum, an important node of the BGTC circuits, has been associated with the number of episodes in MDD after controlling for the effects of medication (Meng et al., 2014). Connectivity deficits in this study mostly involved inferior prefrontal regions whereas the present study found that the medial prefrontal connectivity between the dorsal anterior cingulate cortex, a node more closely associated with directed effort, and the striatum differed between groups. Anomalies in these circuits have also been widely implicated in schizophrenia (Williamson, 2006). Glutamatergic changes in nodes of these circuits correlate highly with social functioning (Aoyama et al., 2011) and resting state connectivity anomalies in the striatum and thalamus have been found in patients with schizophrenia in other studies (Welsh et al., 2010; Anticevic et al., 2014; Wang et al., 2015). An improvement in symptomatology with antipsychotic medications has also been correlated with connectivity between the striatum and the anterior cingulate cortex, dorsolateral prefrontal cortex, and limbic regions (Sarpal et al., 2015). Thus decreased connectivity in patients with schizophrenia compared to MDD patients might be expected in regions associated with this circuit.

Our findings are very similar to those of Ongur et al. (2010) and Chai et al. (2011) who both used a similar seed in the medial prefrontal cortex in chronic schizophrenia and bipolar disorder. In patients with schizophrenia decreased connectivity with the medial prefrontal seed was demonstrated with the dorsal anterior cingulate cortex while bipolar patients demonstrated similar decreased connectivity to the perigenual ventromedial prefrontal cortex, a mood enhancing region which might be expected to be affected in mood disorders (Myers-Schulz and Koenigs, 2012). Findings were also consistent with intrinsic network studies of schizophrenia and bipolar disorder, which showed anomalies in various frontoparietal control networks in schizophrenia and ventral prefrontal emotion encoding regions in bipolar patients (Calhoun et al., 2012; Meda et al., 2012; Anticevic et al., 2014; Baker et al., 2014). While several large-scale networks have been implicated in both disorders, the anterior cingulate cortex (BA32) was found to be one of the few regions which distinguished diagnostic groups (Calhoun et al., 2012).

### Anterior Insulae Seed Connectivity

Seeds in the left and right anterior insula also showed widespread connectivity differences between the patient groups and healthy controls. Compared to healthy controls, patients with schizophrenia had reduced connectivity between the right anterior insula and the right dorsal anterior cingulate cortex as suggested by the Williamson and Allman (2012) model. As hypothesized by the salience models, there was reduced connectivity between the medial prefrontal cortex and both anterior insulae. Patients with MDD demonstrated widespread decreased connectivity between the insulae and frontal, temporal, parietal and posterior cingulate regions compared to controls. Curiously both insular seeds showed similar differences in connectivity to the medial prefrontal seed between depressed patients and patients with schizophrenia involving the dorsal anterior cingulate cortex and regions associated with BGTC circuits but none of these were close to a FDR correction.

Our anterior insulae findings are consistent with recent reports in chronic schizophrenia and MDD. Palaniyappan et al. (2013) demonstrated aberrant connectivity between the right anterior insula and an executive network including the dorsolateral prefrontal cortex in schizophrenia. A number of other studies have confirmed abnormal connectivity between the anterior insula and other regions of the brain in chronic schizophrenia (Mamah et al., 2013; Moran et al., 2013; Orliac et al., 2013; Manoliu et al., 2014b). Abnormal connectivity between the anterior insulae and other parts of the brain have also been found in MDD and bipolar disorder (Mamah et al., 2013; Tahmasian et al., 2013; Manoliu et al., 2014a). These studies did not use a seed-based analysis which might explain why they did not demonstrate differences between BGTC regions.

### Relevance to Large-Scale Network Models

The present study offers a different perspective on the proposed intrinsic networks underlying schizophrenia and MDD. Both the medial prefrontal region and the anterior insulae are associated with widespread differences in connectivity in both disorders compared to controls. Particularly prominent are the deficits in connectivity between both regions and the posterior cingulate cortex in schizophrenia, which might support the model proposed by Buckner et al. (2008). However, deficits are also seen in patients with MDD compared to controls. The suggestion that default regions are highjacked by emotional encoding regions (Northoff et al., 2011) is not supported by this study, which showed decreased connectivity between the medial prefrontal region and ventral prefrontal emotional processing regions.

Models proposed by Menon (2011) and Palaniyappan and Liddle (2012) are supported by the widespread connectivity deficits seen between the anterior insulae and many brain regions in patients with schizophrenia compared to controls. Widespread deficits are also seen in MDD compared to controls suggesting that this may not be specific to schizophrenia. The salience network as suggested by Seeley et al. (2007) includes the anterior insulae and dorsal anterior cingulate cortex. Connectivity with the anterior insulae does not necessarily implicate the salience network. In fact, we did not find clear differences between groups in connectivity between the insulae and the anterior dorsal cingulate cortex, which would be expected if the salience network were involved. Instead our findings suggested that both the medial prefrontal cortex and the anterior insulae demonstrate abnormal connectivity patterns that converge on BGTC circuits.

Both the anterior insulae and the medial prefrontal cortex are highly adapted in the human brain (Craig, 2009; Williamson and Allman, 2011; Uddin et al., 2013). Craig (2009) has suggested that the anterior insula may be a potential neural correlate of consciousness, a suggestion recently supported by the demonstration that the claustrum, which surrounds the insula, is the on/off switch for consciousness (Koubeissi et al., 2014). Williamson and Allman (2011, 2012) have suggested that these unique human capabilities may be related by complex networks associated with connections between Von Economo neurons in the anterior cingulate cortex and anterior insulae and the medial prefrontal cortex. The evolutionary cost of this increasing complexity was vulnerability in the other circuits that control these representational networks, i.e., the directed effort and emotional encoding networks. Failure of the *dorsal* directed effort nodes leads to an inability to realize that a thought or action is internally produced which accounts for delusions of passivity, paranoia and hallucinations in schizophrenia while a failure of the *ventral* emotional encoding nodes leads to the inability to appropriately emotionally respond to current circumstances seen in mood disorders. The present study has provided some support for this view.

### Limitations

A number of limitations must be acknowledged. Patients in this study were receiving medication. Antidepressant medication effects on resting networks are variable (Dutta et al., 2014) but antipsychotic medications could affect BGTC regions (Abbott et al., 2011) accounting for some of the differences between patients with schizophrenia and MDD. However, patients with schizophrenia did not show the same pattern of subcortical differences from healthy controls, suggesting antipsychotic medication alone could not account for the differences found between patients with schizophrenia and MDD. Further, there were no correlations between connectivity and medication dose in patients with schizophrenia. A recent study (Zhang et al., 2015) has also found a marked loss of cortical thickness in the medial prefrontal cortex in chronic schizophrenic patients who have not been treated with neuroleptics suggesting that this region is disconnected from other brain regions. Another recent study (Chung et al., 2015) showed that high-risk patients who converted to psychosis had accelerated medial prefrontal and anterior cingulate cortical gray matter loss. Hence, it seems unlikely that medications could account for all the differences between groups, however, future studies should address drug-naïve patients. It is acknowledged that the patients and healthy controls may have not been entirely at rest although they were instructed to let their minds wander. This is a limitation of all resting state studies but, despite this, there is a fairly consistent literature of identifiable networks in both healthy controls and patient groups, which seems to be independent of differences in level of participation. Other limitations include differences in gender and smoking status between groups but both gender and smoking were covaried. It is of note that smoking may lead to increases rather than decreases in BGTC circuits (Jacobsen et al., 2004).

## Conclusion

Widespread connectivity differences from healthy controls were found in early schizophrenia and MDD using the anterior insular seeds. Using the medial prefrontal seed, differences from healthy controls were also widespread but included the dorsal anterior region in schizophrenia and the ventral prefrontal region in MDD. Schizophrenic patients tended to have reduced connectivity between the medial prefrontal seed and BGTC circuits in early schizophrenia compared to MDD. Results were interpreted to support anomalies in nodes associated with directed effort in schizophrenia and nodes associated with emotional encoding in MDD.

## Author Contributions

JT, RN, EO, RM, NR, JA, and PW designed the study. JP, KF, and PW implemented all data analysis and wrote the manuscript. All authors were involved in manuscript revisions. BS performed subject coordination and clinical data acquisition. RT performed imaging data acquisition. PW is the principle investigator and performed clinical assessments.

## Conflict of Interest Statement

The authors declare that the research was conducted in the absence of any commercial or financial relationships that could be construed as a potential conflict of interest.

## Footnotes

1 http://www-sop.inria.fr/epidaure/Collaborations/IRMf/INRIAlign

2 http://www.fil.ion.ucl.ac.uk/spm

3 http://www.nitrc.org/projects/art
